# The Cognitive Role of the Globus Pallidus interna; Insights from Disease States

**DOI:** 10.1007/s00221-017-4905-8

**Published:** 2017-02-28

**Authors:** M. J. Gillies, J. A. Hyam, A. R. Weiss, C. A. Antoniades, R. Bogacz, J. J. Fitzgerald, T. Z. Aziz, M. A. Whittington, Alexander L. Green

**Affiliations:** 1 Nuffield Department of Surgical Sciences, West Wing, John Radcliffe Hospital, University of Oxford, Oxford, OX3 9DU UK; 2 Nuffield Department of Clinical Neuroscience, West Wing, John Radcliffe Hospital, University of Oxford, Oxford, OX3 9DU UK; 3 Hull York Medical School, University of York, Heslington, York, YO10 5DD UK; 4 Victor Horsley Department of Neurosurgery, Institute of Neurology, Queen Square, UCL, London, UK; 5 Department of Neurosurgery, West Wing Level 3, John Radcliffe Hospital, Oxford, OX3 9DU UK

**Keywords:** Globus Pallidus, Deep brain stimulation, Parkinson’s disease, Dystonia, Cognition

## Abstract

The motor symptoms of both Parkinson’s disease and focal dystonia arise from dysfunction of the basal ganglia, and are improved by pallidotomy or deep brain stimulation of the Globus Pallidus interna (GPi). However, Parkinson’s disease is associated with a greater degree of basal ganglia-dependent learning impairment than dystonia. We attempt to understand this observation in terms of a comparison of the electrophysiology of the output of the basal ganglia between the two conditions. We use the natural experiment offered by Deep Brain Stimulation to compare GPi local field potential responses in subjects with Parkinson’s disease compared to subjects with dystonia performing a forced-choice decision-making task with sensory feedback. In dystonic subjects, we found that auditory feedback was associated with the presence of high gamma oscillations nestled on a negative deflection, morphologically similar to sharp wave ripple complexes described in human rhinal cortex. These were not present in Parkinson’s disease subjects. The temporal properties of the high gamma burst were modified by incorrect trial performance compared to correct trial performance. Both groups exhibited a robust low frequency response to ‘incorrect’ trial performance in dominant GPi but not non-dominant GPi at theta frequency. Our results suggest that cellular processes associated with striatum-dependent memory function may be selectively impaired in Parkinson’s disease even if dopaminergic drugs are administered, but that error detection mechanisms are preserved.

## Introduction

The basal ganglia are a network of subcortical nuclei extensively interconnected with the overlying neocortex, which play an essential role in the control of voluntary movement (Smith et al. 1998; Brown 2003). The nature of this control is still to be fully elucidated and several hypotheses, not necessarily mutually exclusive, have been proposed. A consistent theme is that the basal ganglia optimise motor response to environmental cues to gain maximal sensory reward, or in other words, to minimise the cost/benefit ratio of motor behaviour within the current environment (Bogacz and Gurney 2007). This is a multi-faceted process and a variety of studies suggest different nuclei may play different roles in this process, including learning of action-outcome associations (striatum) (Balleine et al. 2009), signalling the receipt of sensory reward (mesolimbic and striatonigral dopaminergic pathways) (Zaghloul et al. 2009; Gan et al. 2010), reducing the probability of motor error in the context of conflict (subthalamic nucleus) (Zavala et al. 2013), error monitoring (Herrojo Ruiz et al. 2014) and the appropriate scaling of ongoing voluntary movements (minimisation of movement cost) in relation to movement goal (predicted reward) (globus pallidus interna) (Turner and Anderson 2005). The introduction of Deep Brain Stimulation for movement disorders such as Parkinson’s disease and Dystonia have allowed some of these theories to be tested in humans, both by recording the electrophysiology of basal ganglia nuclei whilst subjects perform tasks (Jenkinson and Brown 2011) and by testing the psychophysical effects of DBS therapy (Antoniades et al. 2014).

With regards learning, one hypothesis is that the basal ganglia perform fast directed formation of action-reward associations that, with repetition of the task, train slower Hebbian thalamocortical circuits, the basal ganglia acting as a ‘tutor’ to the cortex (Turner and Desmurget 2010). Supporting this view, lesioning or inactivation of the globus pallidus interna, the main output nucleus of the basal ganglia, is associated with impairment of new motor skill acquisition but not the retention or recall of already-learned skills (Desmurget and Turner 2008). Learning can still take place in Parkinsonian subjects despite degeneration of striatonigral pathways critical in the signalling of the receipt of reward feedback upon motor action; Parkinson’s disease subjects are still able to perform implicit memory tasks with a reduced motor component (Sage et al. 2003). This suggests some basal ganglia-dependent learning functions are dopamine or striatum independent. Pallidotomy-ablation of the globus pallidus interna—is associated with a mild impairment of this faculty despite improved motor symptoms (Sage et al. 2003). In contrast, primary focal dystonic sufferers do not appear to suffer from significant cognitive deficits compared to control subjects despite the manifest motor symptoms of the disease and amelioration by pallidotomy (Jahanshahi et al. 2003).

These observations prompt two questions. First, what is the difference between dystonic and Parkinson’s disease GPi ‘tutor’ signals that largely preserves basal ganglia cognitive function in dystonics? The comparison of neural activity in basal ganglia in these two patient groups is particularly interesting as Parkinson’s disease patients have much larger greater loss of dopaminergic neurons, which are thought to encode information about feedback (Schultz et al. 1997), thus feedback related activity present in dystonic but not Parkinson’s disease patients may be related to dopaminergic modulation. Second, what are the similarities in dystonic and Parkinson’s disease GPi outputs during learning that means Parkinson’s disease subjects still have basal ganglia-dependent learning capacity despite degeneration of the striatonigral pathway?

We use a unique natural experiment offered by functional neurosurgery to attempt to answer these questions. Eight patients undergoing Deep Brain Stimulation of the GPi were studied; five with dystonia and three with Parkinson’s disease. Two of the three PD patients were tested ‘on’ and ‘off’ dopamine medication. Local field potential (LFP) electrical activity was recorded from their indwelling brain electrodes during an onscreen version of the Wisconsin Card Sorting Test called Intra-extradimensional (IED) set shifting (Cantab^®^). During this task, subjects have to make a forced choice between two objects and are provided with feedback indicating a positive (correct choice) or negative (incorrect choice) outcome. ‘Correct’ or ‘incorrect’ depends on a series of rules learnt during the task. We analysed evoked potentials related to the sensory feedback component of the task (consisting of an auditory tone specific to correct/incorrect and also visual feedback). We further analysed these changes in the frequency domain and show results from dystonic and PD patients (on and off medication) as well as dominant and non-dominant GPi.

## Materials and methods

### Patient group

The patient group is described in detail in Table 1. Eight patients (four female, four male) were studied: four with focal dystonia (ages at time of testing 21, 53, 59 and 66 years), one with spasmodic torticollis (aged 65 years), three with idiopathic Parkinson’s disease (ages 44, 55, 66). Seven patients were right handed, one left-handed. No dystonic patients were on anti-dystonic medication at the time of testing [failure to benefit from medication is a major indication for DBS in dystonia (Yianni et al. 2011)]. Two of the three Parkinson’s disease subjects were tested prior to receiving normal dopaminergic medications whilst experiencing ‘off’ symptoms, and subsequently whilst ‘on’ medication. This was not possible in the 3rd subject, where only testing ‘on’ medications was possible. Patients gave informed written consent, the study was approved by Oxfordshire Research Ethics Committee A (Ref 08/H0604/58 & 11/SC/0229) and the study conformed to the Declaration of Helsinki.

**Table 1:** Participants’ characteristics

Patient	Diagnosis	Age at surgery	Age at 1st reported symptoms	Handedness	NART IQ	AMIPB (delayed)	SDMT written/oral
1 (m)	Focal dystonia (neck)	53	15	Right	100	100 (108)	98/110
2 (f)	Focal dystonia (L foot)	21	7	Left	90	94 (100)	96/99
3 (f)	Focal dystonia (cervical)	59	35	Right	n/a	n/a	n/a
4 (m)	Spasmodic torticollis	65	59	Right	95	98 (84)	72 (82)
5 (f)	Focal dystonia (cervical)	66	56	Right	102	114 (117)	91 (102)
6 (f)	Parkinson’s disease	66	52	Right	116	Passed (15)	na/0.00
7 (m)	Parkinson’s disease	44	34	Right	102	Passed (11)	−0.06/0.49
8 (m)	Parkinson’s disease	55	47	Right	103	Passed (9)	n/a

All scores have a mean 100 and SD 15 unless otherwise stated. Performance is classified as: impaired (<69), borderline (70–79), low average (80–89), average (90–109), high average (110–119), superior (120–129), very superior (>130)

*NART IQ* National adult reading test intelligence quotient, *AMIPB* adult memory and information processing battery, *SDMT* symbol digit modalities test, *BFMDRS* Burke–Fahn–Marsden Dystonia Rating Scale, *TWSTRS* Toronto Western Spasmodic Torticollis Rating Scale, *UPDRS* Unified Parkinson’s disease Rating Scale

### Surgery

All eight patients underwent bilateral Globus pallidus deep brain stimulation using a standard technique (Yianni et al. 2011). Medtronic 3387^®^ DBS leads were placed in bilateral posteroventral GPi. Each electrode has four circumferential 1.5 mm electrodes separated by 1.5 mm. A CT head scan was performed to check lead position before recovery from anaesthesia (verified by Image Fusion with the pre-operative MRI). Internalisation of DBS leads and implantation of internal pulse generators normally took place a week later after clinical testing for efficacy.

### Cognitive task

**Fig. 1:**
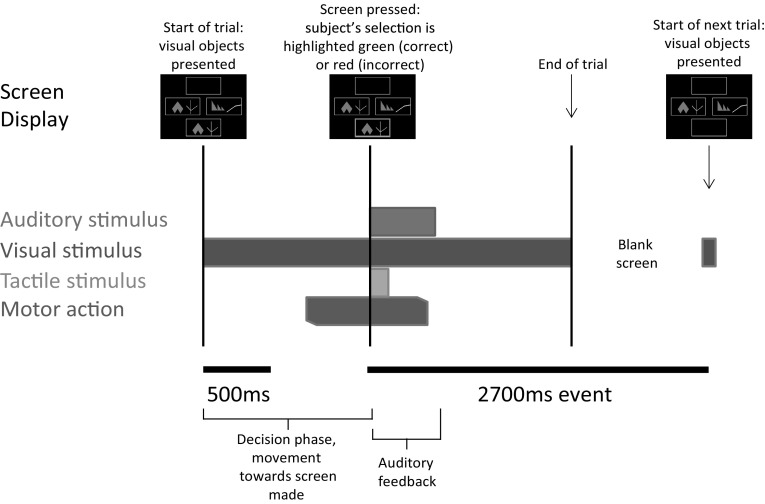
Schematic representations of Intra- Extra Dimensional set shift task rules and events within each trial. **a** Graphic representation of Cantab Intra-extradimensional set shift task rules. See “Materials and methods” for details **b** Graphic representation of sensory and motor events during an individual trial. The trial begins with presentation of the two visual objects. After a variable decision-making phase, the subject then makes a movement to touch the screen. Screen press elicits auditory and visual feedback indicating whether the subject has chosen the correct or incorrect (‘wrong’) figure for the current rule. After an interval of 1.5 s, the screen becomes blank before the start of the next trial.

Subjects performed an on-screen variation of the Wisconsin card sorting test called Intra-extra dimensional (IED) set-shifting (Cantab^®^). This test was used because it is used in clinical practice, and it also has the basic form of object—presentation—motor—action—outcome (feedback)—repeat. The basal ganglia, especially the striatum has been proposed to be involved in the learning of action-outcome associations, therefore, we wished to explore this in the GPi. The task begins with the presentation of two abstract figures. The subject selects one by touching the screen and is informed immediately by auditory and visual feedback if the object was ‘correct’ or ‘wrong’ according to the rule governing object selection (unknown to the subject). Through trial and error by touching the screen and receiving feedback the subject learns the rule. The rule is defined as ‘learned’ when subjects achieve six correct object selections in a row, then the rule changes. At this point, the subject will likely unexpectedly get the next choice wrong and again, by trial and error, must learn the new rule. On some rules this is not associated with a change in stimulus pair on screen (reversal rules i.e. the ‘correct’ object becomes ‘wrong’ or vice versa), but in others it is associated with a new pair of objects, either solid figures alone or solid figures with white line figures superimposed. The rules become more complicated (for example intradimensional shift implies rule change in the same category e.g. solid shapes, whereas extradimensional shift implies a new rule involving the other category i.e. lines). However, in this study, we were primarily concerned with whether a response was ‘correct’ or ‘wrong’ and whether ‘expected’ (i.e. a guess) or ‘unexpected’. The subject must learn 9 rules, with a maximum of 50 attempts allowed per rule otherwise the subject fails the task. (see Fig. 1 for an illustration of the task and timings).

The dystonic patients performed one IED task each. Four of the five dystonic patients passed the test, the subject who failed learned seven rules successfully. Two Parkinson’s disease patients performed one IED task ‘on’ medication and one IED ‘off’ medication. ‘Off’ medication, one subject passed and one failed. ‘On’ medication, both subjects passed. One subject performed three IED tasks in succession passing 1st and 3rd attempts, failing on the 2nd. The Parkinson’s disease patients also performed IED task as part of neuropsychological investigation several months prior to surgery.

### Electrophysiology and analysis

Differential recordings were made from adjacent circumferential 1.5 mm contacts of each deep brain macroelectrode in a bipolar configuration to limit the effects of volume conduction and limit spatial resolution of recordings to a few millimetres of adjacent tissue (Lempka and McIntyre 2013). Globus pallidus contacts were identified by postoperative image-fused MRI and CT. Signals were high pass filtered at 0.5 HZ, amplified (10,000×) using isolated CED 1902 amplifiers and digitised using CED 1401 Mark II at a rate of 2.5 kHz (Cambridge Electronic Design), or recorded via a Porti system (Twente Medical Systems international, B.V., Netherlands) and recorded onto disc using Spike2 software (Cambridge Electronic Designs, Cambridge, UK). Raw data was notch filtered at 50, 100 and 150 Hz as required using Spike2 infinite impulse response Bessel filters, *Q* value adjusted to avoid unwanted filtering of adjacent frequencies as much as possible.

Pre-processing and analysis of LFPs was performed offline using Matlab software and EEGlab (Delorme and Makeig 2004).

Spike 2 data were imported into EEGlab. Raw data were resampled at 300 Hz. 5 s epochs (beginning 2000 ms prior to the start of auditory feedback continuing to +3000 ms) were extracted from left and right GPi contacts and divided into correct and incorrect trials, non-dominant and dominant GPi data as appropriate (see Fig. 1). Trials were divided into correct trials and incorrect trials only; there were insufficient trials to analyse differences between responses to specific rules, e.g. comparison of intradimensional shift to extra dimensional shift responses. Baseline prior to feedback (−2000 to 0 ms) was subtracted, then data were normalised by individual mean and sample standard deviation using Matlab z-score command to allow comparison between different subjects and conditions. EEGlab commands were used to generate event-related potentials, power spectra, event-related spectral perturbations and inter-trial coherence results.

### Statistical analysis

EEGlab non-parametric permutation statistics with False Discovery Rate (FDR) correction were used to compare data between trials and between study groups. The statistical process is as follows: the difference in mean values between the two groups or conditions is calculated, this is the observed test statistic. Next, the data from the two groups is pooled and divided into two groups in every possible combination and mean differences calculated between the resampled groups. The set of mean differences calculated when the data are resampled in this way is the distribution of possible mean differences if the null hypothesis that there is no differences between the two groups is correct. If the observed test statistic lies out with the middle 95% distribution of resampled mean differences, the null hypothesis can be rejected at the 5% level (*P* < 0.05), FDR correction is necessary because of the large number of comparisons inherent in comparing time/frequency plots. Non-parametric Rank Sum and Kruskal–Wallis tests were used to analyse reaction time (RT) data since reaction time data were not normally distributed. RT time data are, therefore, expressed as median: 25–75% interquartile range. RTs were normalised to compare RTs between subjects and conditions in novel trials (rules 3, 4, 6, 8), reversal trials (2, 5, 7, 9) and 1st correct vs 6th correct RTs.

## Results

### Task performance

330 correct trials and 108 incorrect trials in five dystonic subjects were available for analysis, compared to 357 correct trials and 118 incorrect trials in three Parkinson’s disease subjects on medication, and 139 correct and 40 incorrect trials in two Parkinson’s disease subjects off medication. Each dystonic subject performed one IED task each, four passed and one subject failed (failed to acquire 8th rule). Two Parkinson’s disease subjects performed one IED task each on medication, which they both passed, and one off medication (one pass and one fail). One Parkinson’s disease subject performed three IED tasks. This subject failed one (failed to acquire 5th rule) and passed two IED tasks.

Detailed reaction time (RT) data were available from all eight subjects. The reaction times between dystonic and Parkinson’s disease subjects were not directly comparable, especially in the on-medication situation, since the Parkinson’s disease subjects had performed the task on one or more occasions prior to recording, in contrast to dystonic subjects. Reaction times were not significantly different between correct and incorrect trials in dystonic subjects [correct 1484 ms:1202–1966 ms, incorrect 1493 ms:1099.75–2326.25 ms]. Parkinson’s disease subjects on medication demonstrated significantly slower reaction times in incorrect trials compared to correct trials [correct 1181 ms:858–1960.5 ms, incorrect 2112 ms:1355.75–2917.5 ms, *P* < 0.01, Rank Sum test]. Two Parkinson’s disease subjects tested off medication did not demonstrate this difference [correct 1009 ms:859.5–1210 ms, incorrect 1065.5 ms:809.75–1160 ms *P* = 0.98, Rank Sum test]. Off medication, the Parkinson’s disease subjects performed the task significantly faster than on medication (*P* < 0.01, Rank Sum test), despite having performed the off medication IED task prior to on medication task.

Dystonic subjects performed the majority (54%) of incorrect trials during extra-dimensional shift rule 8. Dystonics made significantly fewer errors during intradimensional shift rule 6 compared to extradimensional shift rule 8 [*P* < 0.01, Kruskal Wallis test]. Incorrect trials were more evenly distributed across rules in Parkinson’s disease subjects, with no statistical differences between error rates in the intradimensional and extradimensional shift rules [*P* > 0.01, Kruskal Wallis test].

Dystonic and Parkinson’s disease subjects demonstrated similar prolonged RT responses to novel stimuli (rule 3, 4, 6, 8) trials [*P* < 0.01, Kruskal Wallis test] but were not significantly different from each other in their responses to novel stimuli. Post error (incorrect) trial RT was not significantly prolonged in either group [*P* > 0.01, Rank Sum test], and neither were RTs during the first or second attempts of a reversal rule trial sequence. RT did not change significantly between the first and 6th correct trials of a sequence of six correct trials.

### Electrophysiology

**Fig. 2:**
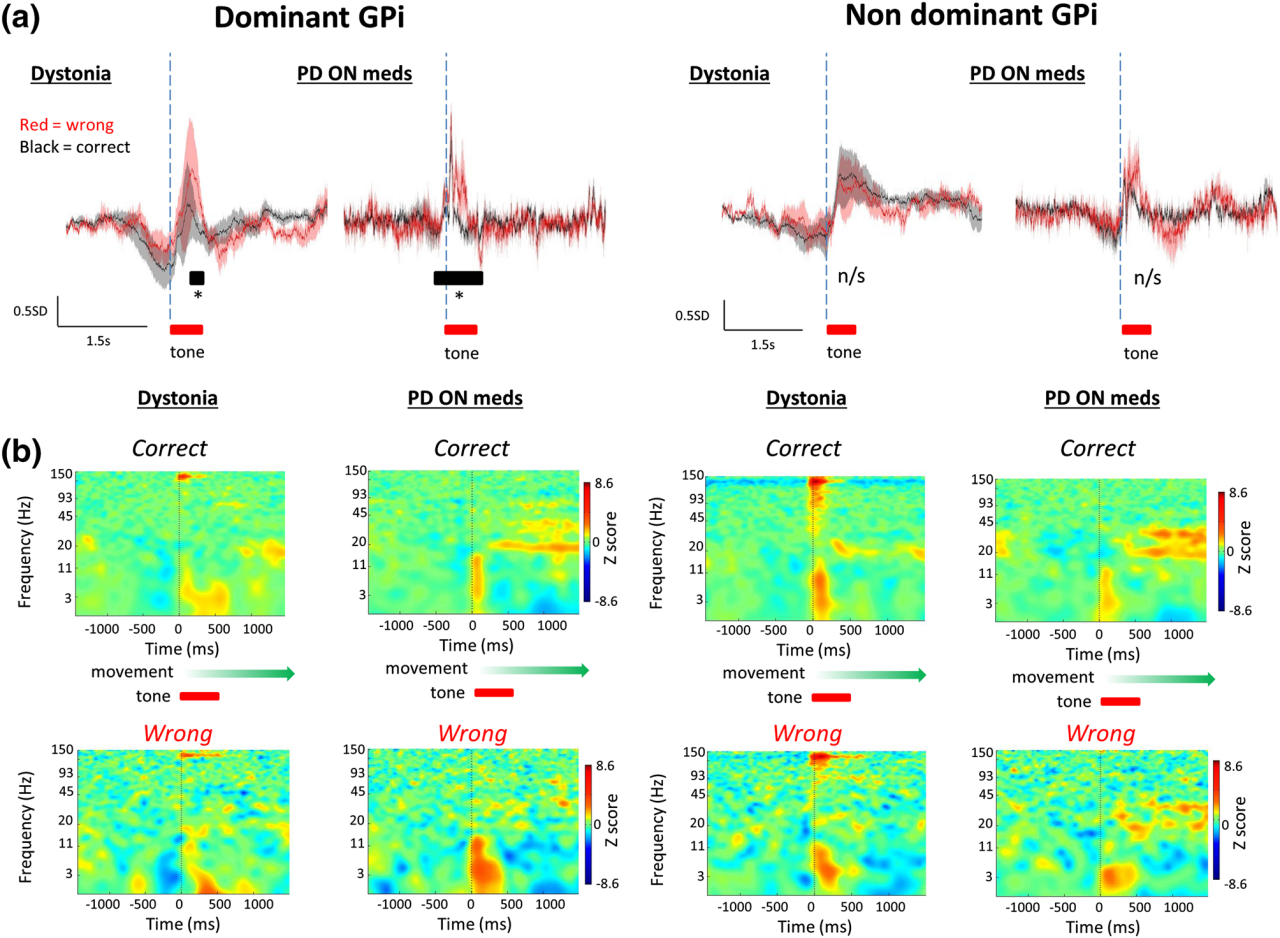
Event-related potentials are elicited in GPi during a forced decision-making task requiring motor output. **a** Coherently averaged normalised ERPs from five dystonic subjects and three Parkinsonian subjects ON medication averaged relative to onset of auditory feedback and grouped according to correct (*black line*) versus incorrect (*red*) trial performance, and dominant vs non-dominant GPi response (mean ± SEM). Movement was associated with a slow negative-going potential in dystonic GPi, peaking and subsequently inverting prior to the commencement of auditory feedback (*hatched line*). Post feedback positive ERP response had significantly greater power in the lower frequency range (<5 Hz) during incorrect trials post feedback cf. correct trials in dystonic subjects in the dominant GPi (*P* < 0.05, permutation + FDR) but not non-dominant GPi. Parkinson’s disease ON medication dominant GPi ERP during correct and incorrect trials demonstrated a significantly smaller deflection from baseline potential than dystonic subjects and a shorter positive phase post feedback (*P* < 0.05, permutation + FDR). No significant differences were observed in non-dominant GPi responses in dystonic or Parkinson’s disease ON medication subjects. Post feedback ERP in Parkinson’s disease subjects ON medication exhibited significantly greater power in the theta band (3–8 Hz) during incorrect trials cf. correct trials in dominant GPi but not non-dominant GPi. Post feedback ERP in dystonic subjects demonstrated significantly greater power in the low theta range (<5 Hz) in incorrect trials cf. correct trials [*P* < 0.05, permutation + FDR]. **b** Averaged Event-related Spectral Perturbance (ERSP) was analysed to further define the response to feedback. Receipt of task feedback (correct vs incorrect via tone and on-screen information) was accompanied by a transient burst of high gamma oscillations (100–150 Hz) in dystonic subjects in both correct and incorrect trials in both dominant and non-dominant GPi. Parkinson’s disease subjects did not demonstrate this response. Post feedback gamma (30–100 Hz) and beta (15–30 Hz) oscillations had significantly greater power in Parkinson’s disease subjects ON medication compared to dystonic subjects, but were not significantly different in either group between correct and incorrect trials. *Scale bar* 0.5 standard deviations (mean 0.0), 1.5 s

Movement to screen was associated with a slow negative-going event related potential in the GPi, peaking and subsequently inverting prior to the commencement of auditory feedback in dystonic and PD subjects (Fig. 2). Parkinson’s disease ON medication dominant GPi ERP demonstrated a significantly smaller deflection from baseline potential than dystonic subjects and a shorter positive phase post feedback (*P* < 0.05, permutation + FDR). No significant differences were observed between non-dominant GPi responses in dystonic cf. Parkinson’s disease ON medication subjects. ERPs from dominant and non-dominant GPi were compared between correct vs incorrect trials in Parkinson’s disease and dystonic subjects. In all three Parkinson’s disease subjects, the dominant GPi was left GPi (right handed). Four dystonic subjects were right handed (left GPi dominant) and one subject was left handed (right GPi dominant). Event related potentials and event-related spectral perturbances (ERSPs) to feedback in PD and dystonic subjects are shown in Fig. 2. Dystonic subjects showed a prominent phasic high gamma signal (frequency 125–135 Hz) upon receipt of sensory feedback lasting approximately 100–200 ms. A phasic high gamma signal was not seen in Parkinson’s disease subjects. Both dystonic and Parkinson’s disease subjects exhibited a greater theta frequency response to incorrect feedback compared to correct feedback in dominant GPi but not non-dominant GPi, albeit at a lower frequency in dystonics compared to Parkinson’s disease subjects.

### High gamma oscillations

High gamma activity (100–150 Hz) was studied in more detail. Average ERSPs from dominant and non-dominant GPi in dystonics revealed an increase in high gamma activity upon receipt of feedback in the range of 125–135 Hz. A phasic high gamma signal was not observed in GPi of Parkinson’s disease subjects on or off medication (Fig. 2b). ERSPs in individual dystonic subjects demonstrated a statistically significant increase in power in the high gamma range [*P* < 0.05, bootstrap procedure] associated with sensory feedback in four of five non-dominant GPi of dystonic subjects and in three of five dominant GPi. The duration of this phenomenon was variable between individuals but ERP images reveal the response was consistent from trial to trial. In Parkinson’s disease subjects there were no statistically significant changes in high gamma band power related to sensory feedback in any of the three subjects in either GPi.

**Fig. 3:**
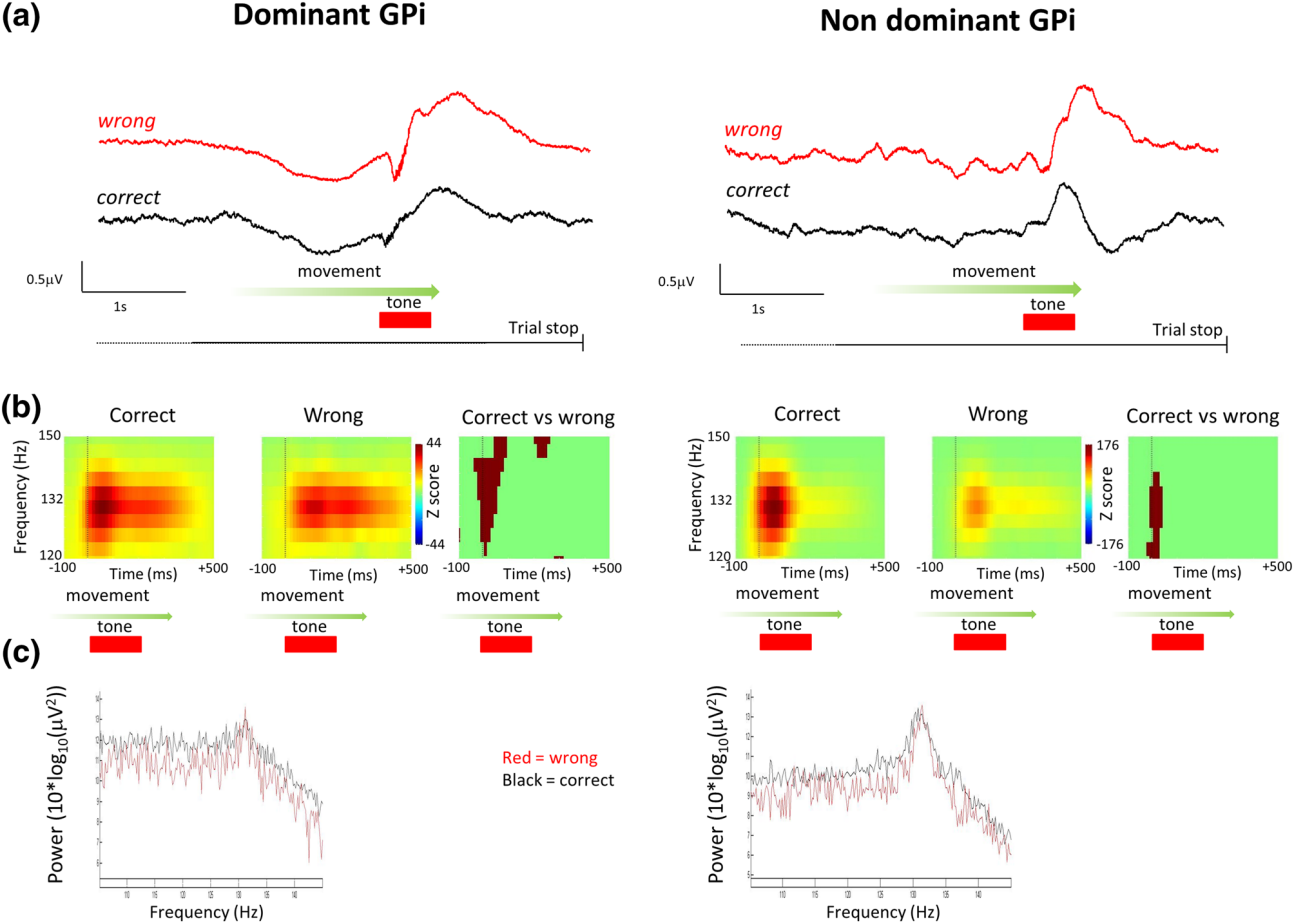
High gamma signal contains trial performance information in dystonics only. **a** Example smoothed ERP from a single patient [patient 2, therefore, dominant side is right GPi]. Onset of auditory feedback is associated with a transient negative deflection nesting a burst of high gamma activity (peak frequency 132 Hz). **b** ERSPs −100 ms prior to start of auditory feedback to +500 ms indicate time to peak high gamma power is longer post start of feedback in incorrect trials cf. correct trials (time to peak correct trials + 20 ms, incorrect trials + 70 ms, *P* < 0.05 permutation stats + FDR) in both dominant and non-dominant GPi. (*n* = 5 subjects, 330 correct trials, 108 incorrect trials). **c** There were no significant differences in high gamma band power between correct and incorrect trials, or between high gamma band power recorded in dominant vs non dominant GPi (not shown)

High gamma oscillations in dystonic subjects occurred nestled on brief negative deflection during the movement related ERP (Fig. 3a). Comparison of high gamma responses between correct trials and incorrect trials indicated that the time to peak high gamma activity from the onset of auditory feedback was significantly longer in incorrect trials compared to correct trials [correct trials time to peak power = 20 ms, incorrect trials time to peak power 70 ms, *P* < 0.05 permutation + FDR] (Fig. 3b). High gamma power was not significantly different when measured across the 3 s peri-feedback epoch between dominant and non-dominant GPi, nor between correct and incorrect trials (Fig. 3c).

### Low frequency responses

Comparing theta frequency (approximately 3–8 Hz) power in correct trials vs incorrect trials in Parkinson’s disease subjects revealed a significantly greater power response in this frequency range upon receipt of sensory feedback in incorrect trials in dominant GPi but not non-dominant GPi between approximately −190 ms prior to the start of sensory feedback to +500 ms [*P* < 0.05, permutation statistics + FDR]. Similarly, there was a significant difference in power at theta frequency between incorrect and correct trials in dystonic subjects on the dominant side only, between +250 and +450 ms post onset of sensory feedback [*P* < 0.05, permutation statistics + FDR]. The theta frequency response had a significantly longer duration in two Parkinson’s disease subjects tested off medication in incorrect trials compared to incorrect trials on medication, and compared to correct trials on and off medication [approx. additional 500 ms, *P* < 0.05 permutation statistics + FDR]. There was no significant difference in theta frequency response on and off medication in correct trials in Parkinson’s disease subjects.

### Beta and gamma band responses

**Fig. 4:**
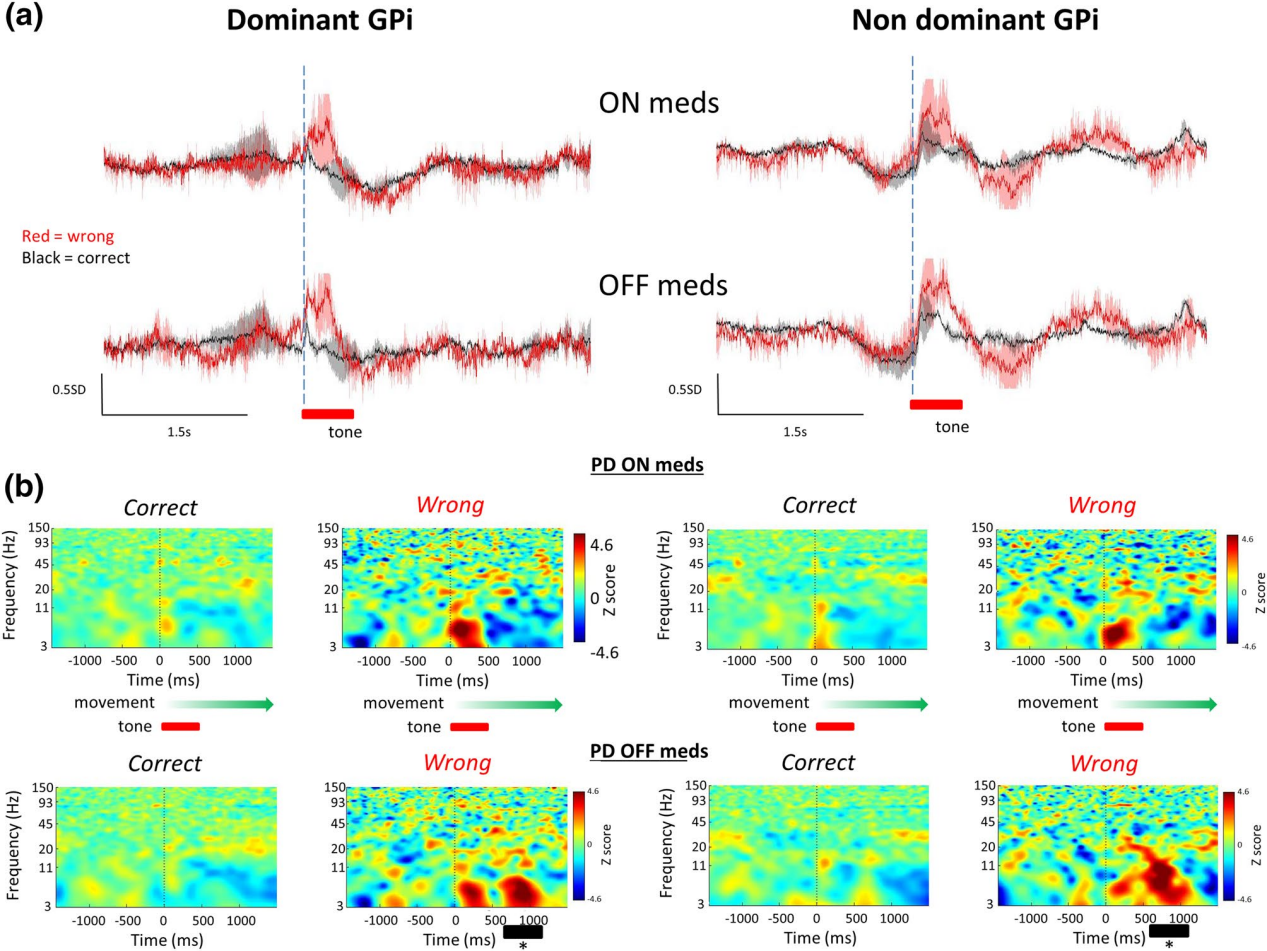
Comparison of ERPs between on medication and off medication conditions in two Parkinson’s disease subjects. Analysis of **a** ERPs and **b** ERSPs in two patients ON and OFF dopaminergic medications (meds), on medication *n* = 118 correct trials, 23 incorrect trials, off medication *n* = 139 correct trials, 40 incorrect trials. Incorrect trials were associated with significantly increased power in the theta band (3–8 Hz) post feedback compared to correct trials both on and off medication. The theta frequency activity persisted longer off medications than on medications. High gamma bursts were not observed in Parkinson’s disease subjects on or off medication in dominant or non-dominant GPi

Beta and gamma frequency bands were then studied. Parkinson’s disease subjects exhibited significantly greater power in both frequency bands in dominant and non-dominant GPi during correct and incorrect trials compared to dystonic subjects [*P* < 0.05, permutation statistics + FDR], as described previously (Ramadan et al. 2009). No sustained statistical differences were found in beta or gamma bands in the peri-feedback time period (0 to +1500 ms) between correct and incorrect trials in dystonic or Parkinson’s disease subjects (see Fig. 4). Analysis of trial by trial ERP responses in individuals demonstrated that beta and gamma band responses were variable from trial to trial in correct and incorrect trials.

## Discussion

### Limitations

This is a rare data set as, in many cases, electrodes are not externalised in Dystonic or Parkinson’s disease patients, dystonia is a rare condition and research historically has focused on motor function rather than cognitive function of GPi. The IED task is complex with potential confounders in interpreting the results such as different forms of rule changes, object variation, changes in stimulus parameters within rules etc. Another confounder is the level of certainty of responses. The unexpected outcomes may be associated with distinct responses that confound the result if unexpected and expected outcomes of the same valence are pooled. The IED test is limited because the certainty of the subject is not assessable in results analysis from trial to trial. However, since valence of the outcome is known trial to trial we are content that pooling correct and incorrect may offer an insight into how the valence of the outcome is represented or processed if details on the magnitude of different types of outcome of the same valence cannot be learned from our data. Our relatively small numbers of trials within the tasks did not allow us to test the effects of these variations in the task, nor could we test other factors for example the effect of different type of rule changes or the difference between intra-dimensional and extra-dimensional tasks. We sought to mitigate against these factors by purely concentrating our analysis on the period immediately before and after feedback. We suppose this period to be most relevant to the aim and seek to mitigate for the variation in object presentation, decision making and variations in movement to screen, since all data were averaged to the onset of (auditory) feedback. We think, therefore, that this study has the strength of indicating the physiology of the GPi immediately before and after feedback in relation to non-motor feedback, and that this design reduces the effect of variables such as visual stimuli presentation, decision making and movement initiation. We propose that our data indicates that GPi plays a cognitive role in cognitive tasks.

### High gamma oscillations

We detected a phasic increase in high gamma oscillations on feedback in globus pallidus interna, in dystonic subjects but not Parkinson’s disease subjects. To our knowledge this is the first description of high gamma oscillations in the globus pallidus. High gamma oscillations, also known as very fast oscillations (VFO), or ‘ripples’, have been studied in the rodent hippocampus, and have been described in human hippocampus and rhinal cortex (Ramadan et al. 2009). They are believed to result from axonal plexus activity amongst gap junction-connected axons of pyramidal cells (Traub et al. 2002), in combination with interneuronal activity (Klausberger and Somogyi 2008). Sharp wave-ripple complexes, similar in morphology to high gamma activity we demonstrate here, have been associated with the formation of long-term memory in neocortex from transient hippocampal based memory. In rodent hippocampus, long-term potentiation, a neurophysiological correlate of memory at a synaptic level, is associated with generation of sharp wave—ripple complexes (Behrens et al. 2005).

Lega et al. detected high gamma oscillations human nucleus accumbens (ventral striatum) in a subject undergoing DBS for depression during a motor task with visual feedback consisting of positive, neutral or negative visual stimuli dependent on motor performance (Lega et al. 2011), and during performance of motor actions requiring enhanced cognitive control (Dürschmid et al. 2013). Our findings echo these findings in that the nature of the sensory feedback appeared to modify the temporal properties of the high gamma oscillation; in nucleus accumbens, high gamma activity occurred earlier in response to positive sensory feedback compared to negative feedback, and high gamma activity occurred at the peaks of on-going alpha activity during positive feedback and at the trough of alpha activity during negative feedback, i.e. a 50 ms phase shift. Our findings suggest that action-outcome information represented in the striatum is transmitted to the GPi in the presence of normal striatonigral pathway function. The relative timing difference between high gamma oscillations in correct vs incorrect trials raises the possibility that the high gamma signal is a representation of axonal discharge via the direct and indirect pathways, respectively, but we cannot verify this. The absence of a phasic high gamma signal in Parkinson’s disease subjects on and off medication suggests this signal relies on intact striatonigral function and is compatible with the idea that the signal originates from the striatum although this would require more detailed study. However, the high gamma oscillation occurs rapidly after the onset of auditory feedback, before the peak of human striatonigral cell firing in response to positive feedback which would contradict this assertion (Zaghloul 2009). Nonetheless, this study was conducted in Parkinson’s disease subjects undergoing DBS surgery; therefore, the applicability of the findings in a non-degenerated striatonigral pathway is not certain. These high gamma oscillations may be the cellular network correlate of the striatum-dependent process of action-outcome association underlying certain forms of cortico-basal ganglia motor learning, and our results imply that this process is impaired in Parkinson’s disease but not dystonia.

### Theta frequency findings

Our findings concerning theta band activity suggest that error detection mechanisms may be conserved in Parkinson’s disease, and are dopamine-independent. Theta oscillations have been reported previously in human subjects, in subthalamic nucleus (STN) and GPi, during variations of the Flanker task (Zavala et al. 2013; Herrojo Ruiz et al. 2014). In STN, theta oscillations have been proposed to represent a ‘hold-your-horses’ function in situations of cue conflict, in other words, to increase the threshold for movement in the presence of conflicting cues to reduce the risk of making a movement error. In GPi, on the other hand, theta oscillations have been associated with the detection of upcoming and actual performance of motor error. This error signal precedes the occurrence of cortical error-related negativity, suggesting that the basal ganglia output may drive or contribute to the processing of motor error evaluation by the medial frontal cortex. Our task differed from the Flanker task in that sensory feedback was presented to the subject on actual outcome of the movement, but our results support the view that the basal ganglia participate in the early detection of error. The suggestion that this function of the basal ganglia is dopamine independent implies that error management is mediated by the hyperdirect pathway, avoiding processing steps associated with the striatum.

Our data does raise the possibility that this mechanism is abnormal in dystonia. Theta oscillations did not show anticipation of incorrect feedback in dystonic GPi as they did in Parkinsonian GPi, the frequency of theta oscillations was lower in dystonics cf. Parkinson’s disease, and dystonic subjects’ reaction times were not prolonged in incorrect trials significantly compared to correct trials, in contrast to Parkinson’s disease subjects. This could suggest the ‘hold your horses’ function of the basal ganglia in conflict is disordered in dystonia. However, theta oscillations were significantly more prominent in Parkinson’s disease subject GPi off medication than on medication and in general cognitive deficits are not prominent in dystonia. These findings, therefore, require further investigation. It should be noted that both Parkinson’s disease subjects and dystonic subjects were able to pass the test.

### Applicability of dystonia results to normal basal ganglia function

We propose that our dystonia group is more likely to be representative of normal GPi function since primary dystonia is not associated with neurodegeneration or dopamine-depletion. This conjecture requires further study and validation, not least because a higher failure rate in IED tasks has been reported in dystonic subjects compared to healthy controls (50 vs. 5%), suggesting dystonia may be associated with attentional-executive deficits (Scott et al. 2003). Although four out of five did pass the test, the group did appear to have difficulty with the extra-dimensional shift rule 8 phase of the task. Difficulty with extra-dimensional shift tasks has been reported in Parkinson’s disease sufferers, suggesting basal ganglia function may be impaired to an extent in dystonic subjects too. However, Scott’s study treated multiple forms of dystonia (generalised, genetic, focal, etc.) as one group, the comparison groups were relatively small, therefore, the magnitude of the difference between the two groups may be overestimated, and the findings may be accounted for by factors such as dystonia-associated pain and depression which may have impaired subjects’ performance. Other studies have not found significant deficits in dystonic subjects.

### Laterality differences

Our data suggest that low frequency activity–generating neural networks are more active in response to sensory feedback in the dominant than the non-dominant hemisphere. In contrast, high gamma frequency-generating neural networks are active in both dominant and non-dominant hemispheres. This could suggest that the non-dominant hemisphere basal ganglia may play a role in sensory signalling or memory formation during this form of task whereas the dominant basal ganglia plays more of a role in motor error detection networks, regardless of handedness of the subject. Our data may give a clue to how basal ganglia function differs between dominant/non-dominant basal ganglia, but unfortunately we have insufficient data to analyse these findings in more detail. Dedicated studies comparing right and left handed subjects are required to investigate this further.

## Conclusion

Our results demonstrate that GPi in both Parkinson’s disease and dystonia subjects responded selectively to ‘correct’ feedback and ‘incorrect’ feedback suggesting that outcome valence of motor action is represented in the GPi, especially marked in the theta band in Parkinsonian subjects. Additionally, we found that phasic high gamma oscillations associated with sensory feedback in dystonic subjects were absent in Parkinson’s disease subjects both on and off medication. Moreover, in dystonic subjects there was a difference in the timing of the onset of high gamma oscillations depending on whether the subject had made the ‘correct’ or ‘incorrect’ object choice, echoing a similar finding in ventral striatum (Lega et al. 2011). Our results suggest that there is more than one mechanism contributing to basal ganglia-dependent learning function, allowing the Parkinsonian basal ganglia to still act as a ‘tutor’ to thalamocortical circuits despite striatonigral pathway degeneration. Movement error processing appears to remain intact in Parkinson’s disease subjects. This may be possible because this function is mediated by the hyperdirect pathway, bypassing the striatum. Dystonic GPi, in contrast, may be a better ‘tutor’, since action-outcome association information processed by the striatum seems to be represented in the GPi. Studies in primates indicate that a GPi–habenular pathway is present that signals both positive and negative outcome information (Bromberg-Martin et al. 2010). Our data suggest that both positive and negative outcome signals are present in the GPi, although the specific origin of this signal cannot be confidently determined. In summary, our data offer valuable insights into GPi function in learning and into the cellular network activity consequences of Parkinsonian neurodegeneration.
